# Outcomes of pregnancy in women with different types of pulmonary hypertension

**DOI:** 10.1186/s12872-023-03423-4

**Published:** 2023-08-09

**Authors:** Yang Liu, Haitao Li, Yanna Li, Jun Zhang, Hong Gu, Jiangang Wang, Qiang Wang

**Affiliations:** 1 Department of Pediatric Cardiac Center, Beijing Anzhen Hospital Affiliated to Capital Medical University, Chaoyang District, Beijing, China; 2 Department of Adult Cardiac Center, Beijing Anzhen Hospital Affiliated to Capital Medical University, Chaoyang District, Beijing, China; 3 Department of Obstetrics and Gynecology, Beijing Anzhen Hospital Affiliated to Capital Medical University, Chaoyang District, Beijing, China; 4 Capital Medical University, Beijing Anzhen Hospital, No.2 An Zhen Road, Chaoyang District, Beijing, 100029 China

**Keywords:** Pulmonary hypertension, Pregnancy, Congenital heart disease, Left heart disease, Eisenmanger syndrome, Idiopathic pulmonary hypertension

## Abstract

**Background:**

Pulmonary hypertension (PH) is considered to increase maternal and fetal risk, and we attempt to explore pregnancy outcomes in women with different types of PH.

**Methods:**

We retrospectively analyzed the clinical data of pregnant women with PH who were admitted to Anzhen Hospital from January 2010 to December 2019, and followed up on these parturients and their offspring.

**Results:**

Three hundred and sixty-six pregnant women with PH were collected, including 265 pulmonary arterial hypertension (PAH) associated with congenital heart disease (CHD), 65 PH caused by left heart disease, 12 idiopathic PH, and 24 PH associated with other diseases. Maternal mean age was 28.4 ± 4.4 years and 72.1% were nulliparous. The estimated systolic pulmonary artery pressure was < 50 mmHg in 40.2% of patients, 50–70 mmHg in 23.2%, and > 70 mmHg in 36.6%. In more than 94% of women, a diagnosis of PH was made before pregnancy. During pregnancy, heart failure occurred in 15% of patients. Cesarean section was performed in 90.5% (20.4% emergency). Complications included fetal mortality (0.5%), preterm delivery (40.4%), and low birth weight (37.7%). A total of 20 mothers died (5.5%). The highest mortality rate was found in patients with idiopathic PH (4/12, 33.3%). A total of 12 children died (3.3%), 5 (1.4%) of them after discharge from the hospital, and 7 (1.9%) were in hospital.

**Conclusions:**

Although most of these women are fertile, PH does increase maternal and fetal risk. Women with idiopathic PH and Eisenmenger syndrome are not recommended to have children.

**Supplementary Information:**

The online version contains supplementary material available at 10.1186/s12872-023-03423-4.

## Introduction

Pulmonary hypertension (PH) is a pathophysiological condition that often leads to debilitating symptoms and shortened overall life expectancy, caused by narrowing of the pulmonary vasculature and often leads to right heart failure (HF). The World Health Organization (WHO) ranks PH as a IV risk [1]. The risk of serious cardiovascular events during pregnancy ranges from 40%-100%, which is contraindicated during pregnancy [2]. However, in clinical practice, pregnancy complicated with PH is not uncommon and is an important cause of maternal death. Although there are risks, some women still want to give birth. In order to realize this dream of motherhood, they even disregard the objections of their family members and doctors [3].

In 2007, Beijing Anzhen Hospital affiliated to Capital Medical University was designated as the only referral and consultation center for pregnancy complicated with heart disease in Beijing. Almost all pregnant women with heart disease go to Anzhen Hospital for treatment. Because many women suffer from PH due to heart disease, the number of hospitalized women suffering from PH is increasing year by year.

There have been some previous studies on women with PH, however, owing to the absence of larger (> 20 cases) prospective outcome studies on PH in pregnancy, many questions remain unanswered. Considering that there are many types of PH [4, 5], this study compared and analyzed the complications and outcomes of puerpera with PH in each group to provide more evidence for the clinical management of pregnant women with PH [6, 7].

## Methods

**Fig. 1:**
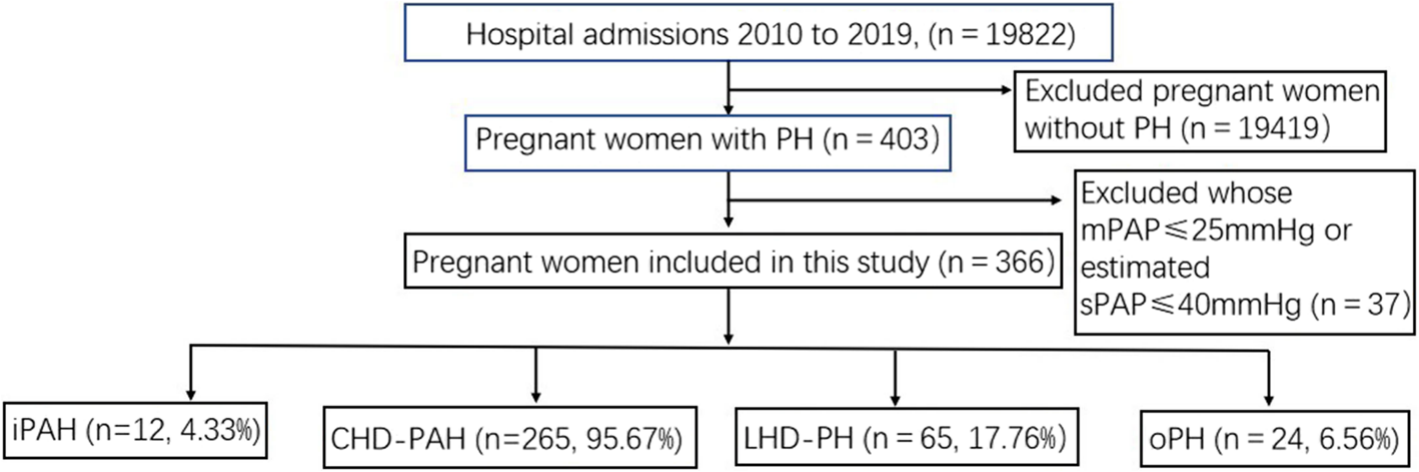
The flowchart of data collection

This study retrospectively analyzed the clinical data of 366 patients with PH who were admitted to our hospital between January 2010 and December 2019 (Table 1 and Fig. 1). This study included patients who were delivered in our hospital and diagnosed by echocardiography as pulmonary artery systolic pressure (sPAP) > 40 mmHg [8]. Patients who were born in other hospitals or did not meet the PH diagnostic criteria were excluded from this study. We divided women with PH into 4 groups: congenital heart disease (CHD-PAH), idiopathic disease (iPH), left heart disease (LHD-PH), and other pH (oPH), and the variable differences of each group were analyzed (Table 2). According to sPAP, patients were divided into 3 groups: mild, moderate, and severe, and the corresponding data were < 50 mmHg, 50–70 mmHg, and > 70 mmHg, respectively. The outcomes of the mothers and fetuses were also analyzed. These women and their offspring were followed up, including gestational age, heart failure, death, general anesthesia, birth weight, preeclampsia, placental abruption, fetal distress and other events.

**Table 1:** Classification of pregnant women with pulmonary hypertension

	n	%
**All PH**	366	
**Aetiology of PH**		
**Group 1 PAH**	277	75.7
Idiopathic PAH	12	3.3
PAH in CHD	265	72.4
ES	59	16.1
left-to-right shunts	138	37.7
Post operative	54	14.8
Other PAH	14	3.8
**Group 2 Left heart disease**	65	17.8
Valvular		
Mitral stenosis	29	7.9
Mitral regurgitation	11	3.0
Aortic stenosis	2	0.6
Other valve disease	1	0.3
Dilated CMP	5	1.4
Hypertrophic CMP	3	0.8
PPCM	2	0.6
Other CMP	2	0.6
Hypertensive heart disease	9	2.5
c-TGA, with systemic ventricular dysfunction	1	0.3
**Group 3 Other PH**	24	6.6
Connective tissue disease	4	1.1
Other PH	20	5.5

*CHD* congenital heart disease, *CMP* cardiomyopathy, *c-TGA* corrected transposition of the great arteries, *ES* Eisenmanger syndrome, *PAH* pulmonary arterial hypertension, *PH* pulmonary hypertension, *PPCM* peripartum cardiomyopathy

**Table 2:** Baseline characteristics, management and outcome of pregnant women with PH

Baseline characteristics	PH *n* = 366	Group 1		Group 2	Group 3	*P*-value
		**PAH** *n* = 277		**LHD-PH** *n* = 65	**Other-PH** *n* = 24	
		**CHD-PAH**	**iPAH**			
	n (%)	n (%)	n (%)	n (%)	n (%)	
	366 (100.0)	265 (72.4)	12 (3.3)	65 (17.8)	24 (6.6)	
Age, years (SD)	28.4 (± 4.37)	27.60 (± 4.04)	29.17(± 5.97)	30.54 (± 4.57)	30.96 (± 3.58)	< 0.001
Nulliparous	264 (72.1)	210 (79.2)	3 (25.0)	38 (58.5)	13 (54.2)	< 0.001
Diagnosis made						0.001
Before pregnancy	345 (94.3)	252 (94.7)	9 (75.0)	62 (95.4)	22 (91.7)	0.098
During pregnancy	21 (5.7)	13 (4.9)	3 (25.0)	3 (4.6)	2 (8.3)	0.185
NYHA class						< 0.001
I	42 (11.5)	27 (10.2)	0 (0.0)	7 (10.8)	8 (33.3)	
II	199 (54.4)	156 (58.9)	2 (16.7)	31 (47.7)	10 (41.7)	
III	83 (22.7)	52 (19.6)	6 (50.0)	20 (30.8)	5 (20.8)	
IV	42 (11.5)	30 (11.3)	4 (33.3)	7 (10.8)	1 (4.2)	
sPAP						< 0.001
40–50(≥ 40, ≤ 50)	147 (40.2)	91 (34.3)	1 (8.3)	39 (60.0)	16 (66.7)	
50–70 (> 50, ≤ 70)	85 (23.2)	68 (25.7)	1 (8.3)	13 (20.0)	3 (12.5)	
70–90 (> 70, ≤ 90)	60 (16.4)	42 (15.8)	3 (25.0)	12 (18.5)	3 (12.5)	
> 90	74 (20.2)	64 (24.2)	7 (58.3)	1 (1.5)	2 (8.3)	
Management						
Delivery, median weeks of pregnancy	36.13 ± 2.90	36.40 ± 2.76	31.17 ± 3.83	35.95 ± 2.43	36.17 ± 3.02	< 0.001
Mode of delivery						0.251
Vaginal	35 (9.6)	30 (11.3)	0 (0.0)	3 (4.6)	2 (8.3)	
CS	331 (90.4)	235 (88.7)	12 (100.0)	62 (95.4)	22 (91.7)	
Emergency CS	75 (20.5)	39 (52.0)	5 (6.7)	22 (23.9)	9 (12.0)	< 0.001
General anaesthesia	9 (2.5)	3 (33.3)	0 (0.0)	5 (55.6)	1 (11.1)	0.019
Outcome						
Maternal death	20 (5.5)					< 0.001
During pregnancy	1 (0.3)	1 (0.4)	0 (0.0)	0 (0.0)	0 (0.0)	
Postpartum (< 1 week)	15 (4.1)	11 (4.2)	4 (33.3)	0 (0.0)	0 (0.0)	
Postpartum (> 1 week; < 6 months)	3 (0.8)	3 (1.1)	0 (0.0)	0 (0.0)	0 (0.0)	
Postpartum (> 6 months)	1 (0.3)	0 (0.0)	0 (0.0)	0 (0.0)	1 (4.2)	
Other complications						
PHC	20 (5.5)	15 (5.7)	5 (41.7)	0 (0.0)	0 (0.0)	< 0.001
Heart failure	55 (15.0)	37 (14.0)	7 (58.3)	9 (13.8)	2 (8.3)	< 0.001
Arrhythmology	60 (16.4)	37 (14.0)	3 (25.0)	13 (20.0)	7 (29.2)	0.153
Postpartum haemorrhage	47 (12.8)	36 (13.6)	0 (0.0)	7 (10.8)	4 (16.7)	0.482
Hypertension	14 (3.8)	5 (1.9)	0 (0.0)	8 (12.3)	1 (4.2)	0.001
Placenta previa	9 (2.5)	4 (1.5)	0 (0.0)	5 (7.7)	0 (0.0)	0.026
Gestational diabetes	44 (12.1)	25 (9.5)	2 (16.7)	12 (18.5)	5 (20.8)	0.104
Preeclampsia	46 (12.6)	20 (7.6)	1 (8.3)	18 (27.7)	7 (29.2)	< 0.001
Thromboembolic event	2 (0.5)	1 (0.4)	0 (0.0)	0 (0.0)	1 (4.2)	0.097

*CHD-PAH* pulmonary arterial hypertension associated with congenital heart disease, *CS* Caesarean section, *iPAH* idiopathic pulmonary arterial hypertension, *LHD-PH* pulmonary hypertension caused by left heart disease, *NYHA* New York Heart Association, *PAH* pulmonary arterial hypertension, *PH* pulmonary hypertension, *PHC* pulmonary hypertensive crisis, *sPAP* systolic pulmonary artery pressure

All patients were informed about the nature of the study. Patients or the public were not involved in the design, conduct, reporting or dissemination plans of our research.

### Definitions used in this study

PH is defined as the increase of mean PAP ≥ 20 mmHg in invasive measurement at rest [9]. The termination of pregnancy before 28 weeks was abortion and induction of labor, the termination of pregnancy after 29–36 weeks was premature delivery, and the pregnancy after 37 weeks was full term. Less than 2500 g was defined as low birth weight (LBW). The cardiac functional classification is I-IV based on the degree of physical activity, with reference to the WHO revised cardiac functional classification based on the NYHA cardiac functional classification. A diagnosis of pulmonary hypertensive crisis was defined based on PH, a rapid sharp increase in pulmonary artery pressure within a short period, and approaching the limit of or exceeding the baseline systemic circulation pressure and aortic pressure because of various factors, resulting in severe hypocardiac output (cardiac output < 3.5 L/min), hypoxemia (arterial oxygen partial pressure < 60 mmHg), hypotension (blood pressure < 90/60 mmHg), and acidosis (pH < 7.35) [10, 11].

### Statistical analysis

All analyses were performed with SPSS version 19.0 (SPSS Inc., Chicago, IL, USA) and R version 4.0.4 (R Foundation for Statistical Computing, Vienna, Austria, 2021). Normality of continuous data was checked with Kolmogorov–Smirnov tests and presented either as mean ± standard deviation, or as median and first and third quartiles (Q1–Q3) as appropriate. Categorical data were presented as frequencies and percentages, and chi-square tests were used for comparisons. Differences between groups were assessed using Student’s t-tests or, in case of non-normality, using Mann–Whitney tests. We used one-way ANOVA to determine whether there is a difference in the population mean represented by multiple sample means. Differences in categorical variables were assessed by the use of χ2 test or the Fisher’s exact test. The Kaplan–Meier method was used for the survival curve. *P*-values of < 0.05 were considered statistically significant (two-sided test).

## Results

A total of 19,424 deliveries that were performed at Beijing Anzhen Hospital from January 1, 2010, to December 31, 2019, and 366 puerpera with PH were collected, including 277 women with PAH, 65 with LHD-PH, and 24 with oPH (Fig. 1 and Table 1). Maternal mean age was 28.40 ± 4.36 years and 72.1% were nulliparous. The estimated sPAP was < 50 mmHg in 40.2% of patients, 50–70 mmHg in 23.2%, and > 70 mmHg in 36.6%. In more than 94% of patients, a diagnosis of PH was made before pregnancy.

### Analysis of complications

In all patients with PH, the incidences of maternal death, HF, PHC, preterm delivery, and infants with LBW were 5.5%, 15.0%, 5.0%, 74.6%, and 78.0%, respectively. There were significant differences in events such as maternal death, preeclampsia, HF, and PHC between the groups. PHC, HF, hypertension, placenta previa, and preeclampsia were significantly higher in the iPAH group than in the other groups (*P* < 0.05). There were no differences in the events of arrhythmia and postpartum hemorrhage; more details are shown in Table 2.

### Management

Depending on the severity of PH, one or more targeted drugs to reduce PH were prescribed, including sildenafil, tadalafil, vantavir, remodulin, and even nitric oxide (NO). Diuretics, digoxin and vasodilators were administered to treat cardiac insufficiency during pregnancy. According to cardiac function, vasoactive drugs such as dopamine, dobutamine, epinephrine were used during perinatal period. Extracorporeal membrane oxygenation (ECMO) was used in 3 cases due to postoperative PHC and HF. Unfortunately, all patients died of multiple organ failure.

### Maternal outcomes

Almost all patients required hospitalization, of which 124 cases (33.9%) were hospitalized more than once. The cardiac cause of admission was observed in 109 patients. The median time of admission was 26 weeks (Q1–Q3 = 18.7–34.0). Admission for cardiac issues occurred at a median of 24.6 weeks (Q1–Q3 = 18.5–30.4) and predominantly HF (55 patients, 50.5%). 20 cases (5.5%) died, of which 8 cases (2.2%) died during follow-up and 12 cases (3.3%) died in hospital. 16 parturients (4.4%) died within 1 week of delivery and 4 of 350 parturients (1.1%) died more than 1 week after delivery. 15 (5.7%) maternal deaths were caused by CHD-PAH, 4 (33.3%) by iPAH, and 1 (4.2%) by oPH. Of the 15 maternal deaths associated with CHD-PAH, 1 (1.7%) was L-R-PAH, 10 (16.9%) were ES, 2 (3.7%) were PAH after repair, and 2 (14.3%) were other maternal deaths. The highest mortality was iPAH (4/12, 33.3%) and the second highest was ES-PAH (10/59, 16.9%) in CHD-PAH group. The mean sPAP of 20 dead cases was 103.2 ± 21.1 mmHg, including 19 cases (19/20, 95.0%) with NYHA functional III-IV before delivery, and most of them died from HF, PHC, and multiple organ failure. The details of maternal mortality are presented in Supplementary Table 1. The highest mortality was found in patients with iPAH (although there was a limited number of patients: 4/12, 33.3%). HF (41.7%) and PHC (41.7%) were also higher than other groups, In all patients with CHD-PAH, the incidence of adverse events such as mortality (5.7%), HF (14.0%), PHC (15.7%), preterm delivery (35.8%), and infants with LBW (35.1%) were very high.

**Fig. 2:**
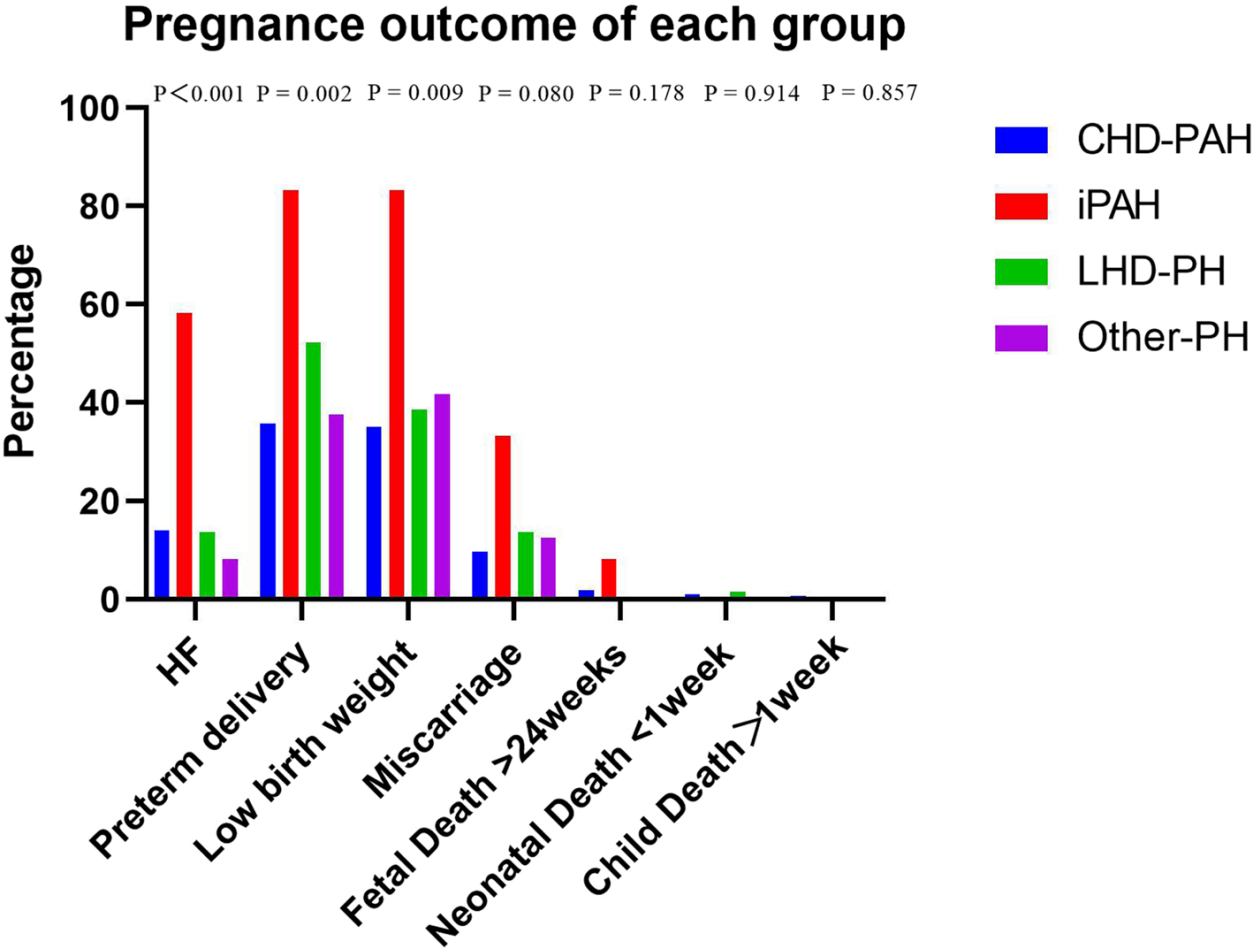
Pregnancy outcomes of different groups

Figure 2 shows the outcome of pregnancy per PH group; HF, preterm delivery, and LBW were significantly different among the 4 groups.

### Delivery

The vast majority of deliveries were cesarean sections (CS, 331, 90.4%), mainly for cardiac reasons (85.6%), and 20.44% were emergency deliveries; all of iPAH were CS. Nine (2.5%) were under general anesthesia during CS. Besides general anesthesia, anesthesia methods for other parturients include epidural, spinal cord, combined epidural, general and local anesthesia.

### Fetal and neonatal outcome

Preterm birth (< 37 weeks), mean neonatal weight, and LBW (< 2500 g) showed marked differences in fetal and neonatal outcomes. iPAH had a lower birth weight and higher incidence of preterm labor and LBW.

A total of 12 children died during the follow-up period, of which 7 died in the hospital and 5 died outside the hospital. The causes of death were associated with preterm infants, low body weight, and hypoplasia. 1 infant, born at week 29, weighed 645 g at birth, 2 were stillborn, 2 subsequently died of lung disease, 1 suffered from multiple malformations and 6 died of premature delivery, low weight and multiple organ dysfunction. Of the 20 mothers and 12 children who died, 6 included children and mothers.

A total of 7 offspring had congenital heart disease (2 ventricular septal defect [VSD], 1 VSD + atrial septal defect [ASD], 1 patent foramen ovale [PFO], 1 patent ductus arteriosus [PDA], 1 partial anomalous pulmonary venous connection + ASD, and 1 tetralogy of fallot). Details of the fetal and neonatal outcomes are presented in Table 3.

**Table 3:** Fetal and neonatal outcome

Fetal complications	PH *n* = 366	Group1		Group2	Group3	*P*-value
		**PAH** *n* = 277		**LHD-PH** *n* = 65	**oPH** *n* = 24	
		**CHD-PAH**	**iPAH**			
	n (%)	n (%)	n (%)	n (%)	n (%)	
	366 (100.0)	265 (72.4)	12 (3.3)	65 (17.8)	24 (6.6)	
Premature delivery (< 37 weeks)	148 (40.4)	95 (35.8)	10 (83.3)	34 (52.3)	9 (37.5)	0.002
Mean newborn weight	2620.87 ± 733.31	2651.98 ± 734.50	1780.00 ± 917.67	2603.92 ± 565.58	2743.75 ± 807.36	0.001
Total LBW (< 2500 g)	138 (37.7%)	93 (35.1%)	10 (83.3%)	25 (38.5%)	10 (41.7%)	0.009
LBW (1500-2500 g)	101 (27.6)	67 (25.3)	4 (33.3)	22 (33.8)	8 (33.3)	
VLBWI (1000-1500 g)	30 (8.2)	21 (7.9)	5 (41.7)	3 (4.6)	1 (4.2)	
ELBWI (< 1000 g)	7 (1.9)	5 (1.9)	1 (8.3)	0 (0.0)	1 (4.2)	
Mortality	12 (3.3)	10 (3.8)	1 (8.3)	1 (1.5)	0 (0.0)	0.456
Fetal mortality > 24 weeks	6 (1.6)	5 (1.9)	1 (8.3)	0 (0.0)	0 (0.0)	0.178
Neonatal mortality < 1 week	4 (1.1)	3 (1.1)	0 (0.0)	1 (1.5)	0 (0.0)	0.914
Neonatal mortality > 1 week	2 (0.5)	2 (0.8)	0 (0.0)	0 (0.0)	0 (0.0)	0.857
Fetal distress	15 (4.1)	10 (3.8)	1 (8.3)	3 (4.6)	1 (4.2)	0.882
Fetal growth restriction	9 (2.5)	5 (1.9)	1 (8.3)	3 (4.6)	0 (0.0)	0.267
Premature rupture of membranes	21 (5.7)	16 (6.0)	0 (0.0)	3 (4.6)	2 (8.3)	0.747
Miscarriage < 24 weeks	42 (11.5)	26 (9.8)	4 (33.3)	9 (13.8)	3 (12.5)	0.080

*ELBWI* extremely low birth weight infant, *oPH* pulmonary hypertension associated with other diseases, *VLBWI* very low birth weight infant. Other Abbreviations as in Tables 1 and 2

### Follow-up

**Fig. 3:**
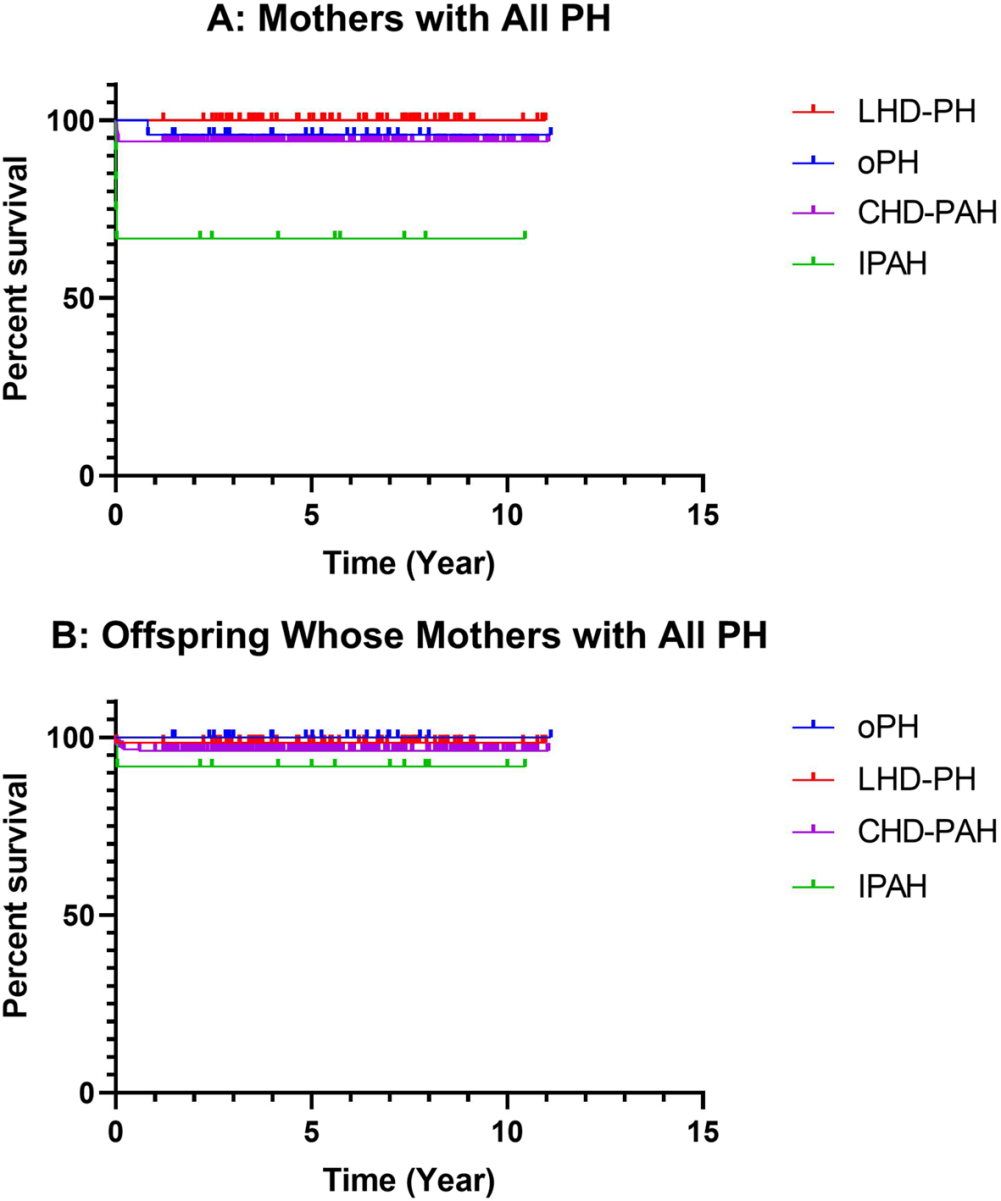
Follow up of mothers and their offspring

A total of 296 patients (80.9%) with PH were followed up, with a mean follow-up of 5.9 ± 2.7 years. A survival curve was observed for the deaths (Fig. 3). There were 36 (12.2%) women who still had symptoms of HF, 42 (14.2%) who still took drugs to reduce PH, and 48 (16.2%) who had limited activity. There was a high incidence of preterm birth and LBW, a high proportion of newborn infants requiring continued treatment, and had problems of slow growth and development at the beginning. However, the growth and development of offspring did not differ from their peers. One offspring's PFO was closed, one child's PDA was recovered, one child's ventricular septal defect was still under observation, and the other offspring with CHD were treated with surgery.

## Discussion

This is the largest series from one institution, where treatment protocols are applied with greater consistency than across multiple institutions. According to our research, the number of women with CHD-PAH was the largest, which was different from the view that LHD-PH is believed to be the most common cause of PH worldwide [12]. One possible reason is that some people with rheumatic heart disease are reluctant to become pregnant after a valve replacement.

Following the previous study by Sliwa et al., mean pulmonary artery pressure (MPAP), when not reported, was approximated or echocardiographic data were utilized using the Bernoulli formula (MPAP = systolic PAP estimated by echocardiogram/4 × 2.4). According to the 2020 ESC Guidelines for the management of adult CHD, PH was defined as an increase in invasively measured mean PAP of ≥ 20 mmHg at rest. We made more rigorous choices, and an sPAP of ≥ 40 mmHg was included in our study.

This study covers almost all the diseases that may cause PH, and major adverse events of these PH parturients, occurred at a much higher rate than in healthy population. Mortality in this group of patients was similar or lower than that previously reported, perhaps because our hospital was characterized by heart disease, our “pregnancy team” was strong and some deaths were avoided. There were marked differences in the maternal and fetal outcomes of women in different groups, and the incidence of arrhythmia was higher in all groups, but there was no significant difference between the groups. The highest mortality was found in patients with iPAH, possibly relating to the unclear pathogenesis, which is consistent with the report of Zhang et al [13]. Combined with the previous studies of our team, the incidence of mortality, HF, preterm delivery, LBW, and other events were indeed higher in CHD parturients with PH than in those without PH (Supplementary Table 2). In terms of fetal outcomes, because of the high incidence of premature delivery (40.4%) and LBW (37.7%), the mortality rate was also high.

The LHD-PH was mostly due to rheumatic valvular disease and may not be as severe in PH as CHD-PAH, so it has a better outcome, which can be seen in the survival curve (Fig. 3). Our research was different from the study by Sadeghi et al., who reported that rheumatoid arthritis-associated PAH patients had comparable survival to iPAH [14].

In all patients with CHD-PAH, the mortality rate was similar to that reported by Ladouceur et al [15]. The adverse events in ES-PAH were more severe, and it is understandable that women with ES should not be allowed to have children. Our view is consistent with that of Li et al. and Duan et al [16, 17], which was also in agreement with the view of Kempny et al [18]. Therefore, the use of surrogate mothers for those with ES was cost-effective and resulted in significantly improved maternal and neonatal outcomes [19]. The incidence of adverse events in post-PAH was lower, which was the same as that reported by Sliwa et al [20].

According to the analysis of sPAP, the higherlevel of sPAP, the higher the risk, which was also in agreement with the view of Miao et al [21]. There was no statistical difference in events such as fetal, neonatal, and child deaths, probably because there was limited data.

Idiopathic PAH is an uncommon but lethal condition, it has a poor prognosis despite available therapeutic options, and the mechanism is not very clear. However, in a recent Japanese study, the survival of Japanese patients with iPAH was good, showing improvement in hemodynamic parameters supported by PH-targeted drugs [22]. There are few large sample studies of pregnant women with iPAH, and some cases have been reported [12, 23]. A study from China reported that maternal mortality of idiopathic PAH parturients was high in a case series. The authors applied epidural anesthesia, early management with multidisciplinary approaches, PAH-specific therapy, avoidance of oxytocin, and timely delivery or pregnancy termination to improve maternal and neonatal outcomes [24].

ES is associated with significant morbidity and mortality in both the mother and the baby. Pregnant women with ES experience hypoxic blood circulation during pregnancy and inadequate placental perfusion, which affects fetal growth and development. Previous studies have suggested that the fetal outcome among mothers with cyanotic heart disease correlated well with maternal hematocrit. Successful pregnancy is unlikely with a hematocrit of > 65%, and over 30% of the fetuses have growth retardation [25]. In our study, the outcomes of both the mother and baby were worse in patients with iPAH and ES than in other groups.

Echocardiography was performed on all patients before and after delivery because it was both effective and non-invasive. Right ventricular systolic pressure was calculated by measuring tricuspid valve regurgitation velocity and right atrial pressure using the simplified Bernoulli equation, as follows: RVSP = 4TRV2 + RAP, right atrial pressure can be estimated by the inferior vena cava diameter and respiratory movement of inferior vena cava. In the absence of pulmonary valve or right ventricular outflow tract stenosis, PASP = RVSP. In the presence of pulmonary valve or right ventricular outflow tract stenosis, PASP = RVSP—differential pressure of pulmonary valve or right ventricular abortion stenosis. The differential pressure of tricuspid valve regurgitation was measured by continuous Doppler, PASP = differential pressure of tricuspid valve regurgitation + right atrial pressure.

Right cardiac catheterization is the gold standard for the evaluation of pulmonary hypertension, but we performed right cardiac catheterization only in few patients with severe PH indicated by echocardiography because it was invasive and expensive. Pregnant women with PH may require general anesthesia to stabilize hemodynamics and prevent PHC and HF [26, 27]. Previous studies have reported a benefit from treatment with PH therapies during pregnancy, including oral sildenafil, tadalafil, and prostanoids. In our study, targeting agents were used in mothers with severe PH, and a combination of more than two targeted therapies is recommended. 3 cases were treated with ECMO for postoperative PHC and HF, and unfortunately all patients died. Undeniably, ECMO remains a life-saving measure in the setting of severe HF, PHC, etc [28–30].

In the follow-up of mothers, the perinatal period (28 weeks of pregnancy to 1 week after birth) was the period with the highest mortality, PHC and other risks. Once the perinatal period was successfully passed, pulmonary artery pressure and cardiac function will improve. The outcome of parturients with L-R PH and their children were good. In addition, in a few parturients with PH during pregnancy, the sPAP returns to normal after delivery. However, mothers with iPAH and ES still suffer from HF, and limited activity and needed medical treatment after childbirth. In the follow-up of the offspring, they experienced LBW, premature deliveries, need for additional care, and even death. Fortunately, after surviving, they gradually caught up with their peers in growth and development.

According to our research, not all mothers with PH can not have children. However, a pregnancy heart group and multidisciplinary managements of pregnant women with PH are imperative. Cardiologists, cardiac surgeons, cardiorespiratory surgeons, pediatricians, anesthesiologists, surgical intensive care unit physicians, respiratory physicians, clinical geneticists, social workers, and psychologists should be included in addition to obstetricians and gynecologists. Proper care is imperative, and pre-pregnancy consultation must be conducted by experienced cardiologists, with detailed clinical evaluation of the parturient and the current hemodynamics. The team should monitor all patients with at least moderate to severe PH before pregnancy, in order to provide timely advice and suggestions during pregnancy, so as to plan prenatal care, including delivery and postpartum follow-up, and the need for heart monitoring. In our research, pregnant women with severe PH need to be discussed by multidisciplinary experts in order to seek better benefits for critically ill women.

In our experience, pregnancy with mild to moderate pulmonary hypertension does not require targeted drug treatment. For the treatment of severe PH, continuing sildenafil and other phosphodiesterase inhibitors, such as tadalafil and vardenafil, is recommended. We do not recommend bosentan or any other endothelin receptor antagonist, such as anlishengtan and masittentan, during pregnancy because of their teratogenic effects. We have the successful use of remodulin during pregnancy. Inhaled nitric oxide for patients with PH has been considered safe, especially during the perinatal period.

Overall, although pregnancy among women with PH is associated with risk and maternal mortality, the majority of these women are fertile. Different types of PH have different outcomes: it was relatively better in parturient with L-R-PAH, LHD-PH, post-PAH, and much more serious in iPAH and ES-PAH. The offspring of pregnant women with PH had a higher risk of premature delivery, LBW, mortality and prenatal monitoring. Women with PH need antenatal counseling and a "pregnancy team" is necessary [31, 32].

### Study limitations

The present study had several limitations. First, it was a retrospective observational single-center study, limiting the generalizability of these results. Second, PH in most parturients was diagnosed by echocardiography, not right cardiac catheterization. As others have emphasized, echocardiography has limitations in diagnosing PH relative to right cardiac catheterization. Therefore, the interpretation of our data must take into account the limitations of echocardiography as a diagnostic method in PH. Third, for privacy reasons, some follow-up information and results provided by individual family members may be inaccurate. 

## Conclusions

The higher the PH, the greater the risk and the different types and degrees of PH will vary. PH caused by CHD is the most common type of PH in pregnant women in China, which increases the risk of childbirth, but the vast majority of them are fertile. Maternity with ES or iPAH is not recommended. Pregnant women with severe PH face issues such as medication and limited postpartum activity. Women with severe PH require counselling before conception and regular post-pregnancy tests such as echocardiography, and very close expert follow-up at least once a month. Perinatal conditions in mothers with PH are associated with issues such as premature delivery, low birth weight, and rescue. The offspring of PH face developmental delay and growth restriction in the early stage, and fortunately, the long-term follow-up results are good.

### Supplementary Information

**Additional file 1:** **Supplementary Table 1.** Death cases of mothers.

**Additional file 2: Supplementary Table 2.** Adverse cardiovascular, obstetric, and fetal events experienced by women with CHD admitted for delivery by presence of PH.

## Data Availability

Data sets generated and analyzed for the current study will be available from the respective authors on reasonable request. Please contact professor Yang Liu if necessary, his email address is: liuyang2010strive@163.com.

## Abbreviations

CHD: Congenital heart disease
CHD-PAH: Pulmonary arterial hypertension associated with congenital heart disease
CS: Caesarean section
ECMO: Extracorporeal Membrane Oxygenation
ES-PAH: Pulmonary arterial hypertension associated with congenital heart disease that progressed to Eisenmenger Syndrome
ES: Eisenmenger syndrome
HF: Heart Failure
iPAH: Idiopathic pulmonary arterial hypertension
LBW: Low birth weight
LHD: Left heart disease
LHD-PH: Pulmonary hypertension caused by left heart disease
L-R-PAH: Pulmonary arterial hypertension associated with left-to-right shunt congenital heart disease.
NYHA: New York Heart Association
oPAH: Pulmonary arterial hypertension associated with other congenital heart disease
oPH: Pulmonary hypertension associated with other diseases
PAH: Pulmonary arterial hypertension
PH: Pulmonary hypertension
post-PAH: Pulmonary arterial hypertension associated with postoperative congenital heart disease;
sPAP: Systolic pulmonary arterial hypertension
