# Supplementary material for: Outcomes of pregnancy in women with different types of pulmonary hypertension
Source: BMC Cardiovasc Disord. 2023 Aug 9;23:391. doi: 10.1186/s12872-023-03423-4 (PMC10410774; doi:10.1186/s12872-023-03423-4)
Supplement: Supplementary file 1 — Additional file 1: Supplementary Table 1. Death cases of mothers. [file 12872_2023_3423_MOESM1_ESM.docx]

**Supplementary Table 1.** Death cases of mothers

|  | Age | Diagnosis | Diagnosis  known  before  pregnancy | Medical  history | ABP | Estimated sPAP | Parity | Pre-pregnancy  counselling | NYHA  before  pregnancy | Events  during  pregnancy | Pregnancy  duration  (weeks) | Multidisciplinary discussion before delivery | Delivery | Birth weight | Fetal  status | Timing of  maternal  death | Cause of  death | Vasoactive drugs | PH  treatment | Hospital stays | Hospitalization: 1 Discharge: 2 |
| --- | --- | --- | --- | --- | --- | --- | --- | --- | --- | --- | --- | --- | --- | --- | --- | --- | --- | --- | --- | --- | --- |
| 1 | 30 | PDA，ES, PHC | Yes | History of induced abortion | 115/61 | 89 | G2P1 | Yes | IV | Pseudoaneurysm of pulmonary artery ruptured ES, HF, pericardial effusion, polyhydramnios | 30 | Yes | Vaginal  delivery | 1040 | Death after 10 days | 9 | Pseudoaneurysm of pulmonary artery ruptured | Dopamine, dobutamine, noradrenaline | Rimoduline, sildenafil | 10 | 1 |
| 2 | 25 | VSD,ES,PH,PHC, MOF, HF | Yes | ES，HF | 132/61 | 100 | G2P1 | Yes | IV | HELLP syndrome, preeclampsia (severe) | 31 | Yes | CS | 1570 | Death after 5 months | 1 | PH，MOF, HF | Dopamine, epinephrine, noradrenaline | NO | 1 | 1 |
| 3 | 20 | VSD,ASD, PDA, ES | No | Restricted physical activity | 135/85 | 107 | G1P1 | No | III | Nephrotic syndrome in pregnancy? ES, PHC | 36 | Yes | CS | 1830 | Death | 2 | PHC, HF, hypoxemia | Dobutamine | Alprostadil, vantavir | 3 | 1 |
| 4 | 28 | VSD, PH, HF, PHC, ES | Yes | HF，ES | 124/75 | 116 | G3P1 | Yes | IV | HF，PHC，Postpartum hemorrhage | 30 | Yes | CS | 1270 | Death | 3 | PHC,HF | Epinephrine, dopamine, norepinephrine, dobutamine, pituitrin | Alprostadil, sildenafil, rimodulin | 5 | 1 |
| 5 | 23 | VSD, PH, PHC, HF | Yes | Restricted physical activity | 151/102 | 131 | G2P1 | Yes | IV | PHC, MOF, HF, preeclampsia | 33 | Yes | CS | 1450 | Death | 1 | PHC，HF， | Noradrenaline, dopamine | NO, vantaville, treprostinil | 3 | 1 |
| 6 | 21 | DORV, VSD, PHC, PH, Pulmonary infection, ITP | Yes | Blood transfusion treatment because of Idiopathic thrombocytopenia purpura | 130/72 | 106 | G1P1 | Yes | IV | PHC，ES，anemia，preeclampsia, fetal distress | 31 | Yes | CS | 1380 | Death | 1 | PHC，HF， | Epinephrine, dopamine, dobutamine, norepinephrine, pituitrin | Sildenafil, rimodulin | 3 | 1 |
| 7 | 29 | PHC，ES, HF, HF, RF, VSD，PH, Sudden death | Yes | ES | 104/62 | 83 | G1P1 | Yes | II | Sudden death, HF, RF, Postpartum hemorrhage, PHC | 32 | Yes | CS | 1560 | Alive | 13 | PHC, ES, Sudden death, HF, RF | Noradrenaline, dopamine | Sildenafil, vantaville | 23 | 1 |
| 8 | 24 | PDA, ES | Yes | ES | 118/78 | 102 | G2P1 | Yes | IV | Postpartum hemorrhage, PHC | 30 | Yes | CS | 930 | Alive | 4 | PHC | Noradrenaline, pituitrin, dobutamine | Tadalafil, rimodulin | 4 | 1 |
| 9 | 27 | VSD, PH, PHC, HF | Yes | History of VSD operation | 124/74 | 121 | G1P1 | No | IV | PHC, HF, Metabolic acidosis, Fetal distress, premature rupture of membranes | 35 | Yes | CS | 2140 | Alive | 1 | PHC, HF, Metabolic acidosis | Dopamine, dobutamine, noradrenaline | Alprostadil rimodulin, sildenafil | 1 | 1 |
| 10 | 27 | VSD，PDA, PH | Yes | History of VSD＋PDA operation, Restricted physical activity | 113/75 | 122 | G1P1 | Yes | IV | PHC, MOF | 34 | Yes | CS | 2110 | Alive | 2 | PHC, MOF | Noradrenaline, dobutamine, pituitrin, milinone | Remodulin, vantaville | 5 | 1 |
| 11 | 27 | IPAH | Yes | No | 114/66 | 74 | G1P1 | No | IV | PHC, MOF, DIC, HF | 28 | Yes | CS | 1190 | Alive | 3 | PHC, MOF, DIC | Noradrenaline, pituitrin, epinephrine, dopamine | Alprostadil, sildenafil, NO | 9 | 1 |
| 12 | 22 | IPAH | No | No | 120/100 | 102 | G1P1 | No | III | Polyhydramnios, Fetal distress, PHC, HF | 37 | Yes | CS | 2880 | Alive | 2 | PHC, HF | Dopamine, dobutamine, adrenaline | Alprostadil | 2 | 1 |
| 13 | 32 | TECD,PDA, PH | Yes | Restricted physical activity | 122/76 | 53 | G1P1 | No | III | Pulmonary infection, pregnancy with pancreatitis, Postpartum hemorrhage | 36 | Yes | CS | 2950 | Alive | 2 | HF | Unknown | Unknown | 4 | 2 |
| 14 | 28 | ASD, PH | Yes | Diagnosis of ASD 2 years ago, no surgical opportunity, recommended termination of pregnancy | 132/106 | 110 | G1P1 | Yes | IV | HF | 33 | Yes | CS | 1950 | Alive | 2 | HF | Epinephrine, norepinephrine, dobutamine, pituitrin | Alprostadil, vantavir, sildenafil | 1 | 2 |
| 15 | 20 | PDA, ES, PH | Yes | Restricted physical activity, PDA occlusion failed because of PH 6 years ago | 168/112 | 116 | G2P1 | No | III | PHC，HF，MOF，ES，preeclampsia，HELLP syndrome | 32 | Yes | CS | 1160 | Alive | 1 | PHC，HF，MOF | Dopamine, norepinephrine, pituitrin | Remoduline | 2 | 2 |
| 16 | 28 | VSD, ES, HF | Yes | Restricted physical activity | 145/106 | 120 | G1P1 | No | IV | PHC，ES，HF，MOF，DIC，preeclampsia，hypoxemia，HELLP syndrome | 35 | Yes | CS | 1330 | Alive | 3 | PHC，HF，MOF，DIC | Dobutamine, Orthonephrosis, Pituitrin, Levosimendan | Remoduline, sildenafil, Vantavir | 1 | 2 |
| 17 | 24 | SV, CAT, TAPVC, ASD, PH | Yes | Restricted physical activity | 130/100 | 133 | G2P1 | Yes | III | Fetal distress, fetal growth restriction, paroxysmal supraventricular tachycardia | 34 | Yes | CS | 1190 | Alive | 10 | HF | Dopamine, noradrenaline | Alprostadil | 10 | 2 |
| 18 | 30 | Dextrocardia，CPHD，PH | No | Chronic pulmonary heart disease, right atelectasis, old pulmonary tuberculosis | 120/80 | 89 | G2P1 | Yes | III | Anemia in pregnancy | 37 | Yes | CS | 2400 | Alive | 5year | Hypoxemia | Dopamine, noradrenaline | NO, Vantavir, sildenafil, rimoduline | 19 | 2 |
| 19 | 28 | Idiopathic pulmonary hypertension | No | Syncope once | 113/81 | 119 | G5P3 | No | III | MOF, HF, DIC, PHC | 29 | Yes | CS | 1200 | Alive | 1 | MOF, HF, DIC, PHC | Epinephrine, norepinephrine, dopamine, pituitrin | NO, rimoduline | 2 | 2 |
| 20 | 25 | Idiopathic pulmonary hypertension | No | No | 106/78 | 71 | G5P2 | No | III | PHC, Hypoxic ischemic encephalopathy | 32 | Yes | CS | 2020 | Alive | 5 | PHC | Epinephrine, norepinephrine, dobutamine | Alprostadil, sildenafil | 4 | 2 |

CAT= Common arterial trunk, CPHD=chronic pulmonary heart disease, CS= Caesarean section, IPAH=idiopathic pulmonary arterial hypertension, ITP= Idiopathic thrombocytopenic purpura, MOF =Multiple Organ Failure, RF=Respiratory failure
