# Supplementary material for: Outcomes of pregnancy in women with different types of pulmonary hypertension
Source: BMC Cardiovasc Disord. 2023 Aug 9;23:391. doi: 10.1186/s12872-023-03423-4 (PMC10410774; doi:10.1186/s12872-023-03423-4)
Supplement: Supplementary file 2 — Additional file 2: Supplementary Table 2. Adverse cardiovascular, obstetric, and fetal events experienced by women with CHD admitted for delivery by presence of PH. [file 12872_2023_3423_MOESM2_ESM.docx]

**Supplementary Table 2.** Adverse cardiovascular, obstetric, and fetal events experienced by women with CHD admitted for delivery by presence of PH

|  | CHD With PAH  （n=265） | CHD Without PAH（n=769） | P value | Crude OR  （95%CI） | Adjusted P value | Adjusted OR（95%CI） |
| --- | --- | --- | --- | --- | --- | --- |
| **Obstetric events** | | | | | | |
| Hypertension in pregnancy | 7（2.64） | 16（2.08） | 0.629 | 1.25（0.51-3.07） | 0.724 | 1.19（0.46-3.04） |
| Placenta previa | 6（2.26） | 20（2.60） | 0.295 | 0.56（0.19-1.66） | 0.412 | 0.63（0.21-1.89） |
| Gestational diabetes | 27（10.19） | 112（14.56） | 0.057 | 0.65（0.42-1.00） | 0.123 | 0.70（0.45-1.10） |
| Placental abruption | 2（0.75） | 6（0.78） | 0.945 | 0.95（0.19-4.71） | 0.961 | 0.96（0.18-5.00） |
| Hemorrhage | 35（13.21） | 102（13.26） | 0.884 | 0.97（0.64-1.46） | 0.531 | 0.87（0.57-1.34） |
| Pre-term delivery | 95（35.85） | 77（10.01） | ＜0.001 | 2.84（1.97-4.09） | ＜0.001 | 2.23（1.52-3.29） |
| preeclampsia | 21（7.92） | 47（6.11） | 0.350 | 1.29（0.76-2.20） | 0.879 | 0.96（0.53-1.71） |
| **Cardiovascular events** | | | | | | |
| Heart failure | 34（12.83） | 17（2.1） | ＜0.001 | 6.35（3.48-11.57） | ＜0.001 | 4.95（2.48-9.87） |
| Arrhythmia | 35（13.21） | 97（12.61） | 0.898 | 1.03（0.68-1.55） | 0.873 | 1.04（0.68-1.58） |
| Thromboembolic event  (stroke, PE, and so on) | 1（0.38） | 3（0.39） | 0.961 | 0.95（0.10-9.13） | 0.919 | 1.12（0.12-10.92） |
| **Delivery procedure** | | | | | | |
| Cesarean section | 240（90.57） | 581（75.55） | ＜0.001 | 2.51（1.67-3.77） | ＜0.001 | 2.32（1.53-3.51） |
| Artificial rupture of the membranes | 2（0.75） | 24（3.12） | 0.047 | 0.23（0.05-0.98） | 0.049 | 0.10（0.01-0.77） |
| Induction | 2（0.75） | 24（3.12） | 0.047 | 0.23（0.05-0.98） | 0.049 | 0.09（0.01-0.72） |
| **Fetal events** | | | | | | |
| Fetal distress | 10（3.77） | 48（6.24） | 0.120 | 0.58（0.29-1.15） | 0.117 | 0.46（0.21-1.02） |
| Fetal growth restriction | 4（1.51） | 8（1.04） | 0.566 | 1.43（0.43-4.77） | 0.545 | 1.17（0.32-4.31） |
| Fetal malformation | 1（0.38） | 1（0.13） | 0.460 | 2.84（0.18-45.63） | 0.477 | 2.74（0.17-43.93） |
| Infant of low-birth weight | 91（34.34） | 48（6.24） | ＜0.001 | 2.68（1.72-4.17） | ＜0.001 | 2.40（1.51-3.79） |
| **Other events** | | | | | | |
| Respiratory diseases | 7（2.64） | 7（0.91） | 0.049 | 2.89（1.00-8.31） | 0.048 | 2.94（1.02-8.44） |
| Systemic hypertension | 4（1.51） | 17（2.21） | 0.463 | 0.66（0.22-1.99） | 0.455 | 0.63（0.20-2.12） |

**CHD:** congenital heart disease; **CI:** confidence interval; **OR**: odd ratio; **PE**: pulmonary embolism; **PH**: pulmonary hypertension. Values are n (%) unless otherwise indicated.
